# Heterologous Expression, Purification and Characterization of an Alkalic Thermophilic β-Mannanase CcMan5C from *Coprinopsis cinerea*

**DOI:** 10.3390/jof9030378

**Published:** 2023-03-20

**Authors:** Songling Yan, Baiyun Duan, Cuicui Liu, Guiyou Liu, Liqin Kang, Lei Sun, Lin Yi, Zhenqing Zhang, Zhonghua Liu, Sheng Yuan

**Affiliations:** 1 Jiangsu Key Laboratory for Microbes and Microbial Functional Genomics, Jiangsu Engineering and Technology Research Center for Industrialization of Microbial Resources, College of Life Science, Nanjing Normal University, 1 Wenyuan Road, Nanjing 210023, China; yansongling2023@126.com (S.Y.); dby492991@163.com (B.D.);; 2 School of Life Science and Chemical Engineering, Jiangsu Second Normal University, Nanjing 211200, China; 3 Jiangsu Key Laboratory of Translational Research and Therapy for Neuro-Psycho-Diseases and College of Pharmaceutical Sciences, Soochow University, Suzhou 215021, China

**Keywords:** β-mannanase, alkalic thermophilic β-mannanase, resistance to organic solvents, resistance to detergents, storage stability

## Abstract

A endo-1,4-β-mannanase (CcMan5C) gene was cloned from *Coprinopsis cinerea* and heterologously expressed in *Pichia pastoris*, and the recombinant enzyme was purified by Ni-affinity chromatography and identified by matrix-assisted laser desorption/ionization time-of-flight mass spectrometry (MALDI-TOF/TOF-MS). CcMan5C hydrolyzed only locust bean gum galactomannan (LBG) but not α-mannan from *S. cerevisiae* or Avicel cellulose, oat spelt xylan, or laminarin from *Laminaria digitata*. CcMan5C exhibited distinctive catalytic features that were different from previously reported β-mannanases. (1) CcMan5C is the first reported fungal β-mannase with an optimal alkalic pH of 8.0–9.0 for hydrolytic activity under assay conditions. (2) CcMan5C is the first reported alkalic fungal β-mannase with an optimal temperature of 70 °C for hydrolytic activity under assay conditions. (3) The organic solvents methanol, ethanol, isopropanol, and acetone at concentrations of 10% or 20% did not inhibit CcMan5C activity, while 10% or 20% isopropanol and acetone even enhanced CcMan5C activity by 9.20–34.98%. Furthermore, CcMan5C tolerated detergents such as Tween 20 and Triton X-100, and its activity was even enhanced to 26.2–45.6% by 1% or 10% Tween 20 and Triton X-100. (4) CcMan5C solution or lyophilized CcMan5C exhibited unchanged activity and even increasing activity after being stored at −20 °C or −80 °C for 12 months and retained above 50% activity after being stored at 4 °C for 12 months. These features make CcMan5C a suitable candidate for the detergent industry and paper and pulp industry.

## 1. Introduction

Mannans are one kind of hemicellulose that is mainly present in specialized plant structures such as woods, tubers, seeds, beans, and fruits, as well as fungal and bacterial cell walls [1]. Mannans are divided into α-mannans and β-mannans. β-Mannans have a linear backbone of mannose residues joined by β-(1→4)-mannosidic linkages, and they are further classified into mannan, glucomannan, galactomannan, and galactoglucomannan according to carbohydrate substitutions in the backbone. Of these, galactomannan is the largest group and is composed of a β-(1→4)-linked D-mannan backbone with α-(1→6)-substitutions of D-galactose. The ratios of mannoses to galactoses in galactomannans from different resources vary as locust bean gum (~4:1), tara gum (~3:1), guar gum (~2:1), and fenugreek gum (~1:1) [1,2].

β-Mannans can be hydrolyzed to mannooligosaccharides (MOSs) with various degrees of polymerization (DP, 2–10) and mannose by endo-1,4-β-mannanases (EC 3.2.1.78) [2,3]. Recently, MOSs have been investigated for their in vitro antioxidant activity [4,5], prebiotic activity [3,6,7,8,9] and anticancer activity [4,10,11], and their in vitro and in vivo immunomodulatory activity [12,13,14,15], antiobesity [16,17,18], and antidiabetic activity [19,20,21].

Endo-1,4-β-mannanases attack the internal β-1,4-glycosidic linkages in the β-mannan backbone to release short β-1,4-mannooligosaccharides [22]. A variety of bacteria, fungi, plants, and animals can produce endo-1,4-β-mannanases [23,24,25]. Endo-1,4-β-mannanases are distributed mainly in glycoside hydrolase (GH) families 5 and 26, while some endo-1,4-β-mannanases also are found in GH113 and GH134 according to the Carbohydrate-Active Enzyme Database (http://www.cazy.org/, accessed on 14 March 2023) [26]. Family 5 comprises some bacterial β-mannanases and most eukaryotic β-mannanases, while the β-mannanases in family 26 are of bacterial origin with the exception of a few anaerobic fungi [23]. The primary structures of β-mannanases in different GH families are different but they share similar (b/a)8-barrel folds catalytic domain with two conserved glutamic acid residues, located in the middle of the catalytic core [24,26]. In addition, some β-mannanases contain carbohydrate binding domain(s) (CBD) and additional functional domain(s) [24].

Depending on varying resources and their different amino acid sequences, different endo-1,4-β-mannanases exhibit different catalytic features, resulting in the production of different MOSs from the same galactomannan, such as locust bean gum β-galactomannan (LBG) [4,27,28,29]. In addition, β-mannanases are also applicable and have found applications in different industries such as animal feed, food, biorefinery, textile, detergent, and paper, and pulp [22,23,24,25]. These different application processes require some specific β-1,4-mannanases with distinctive catalytic features. Microorganisms are the main sources of β-1,4-mannanases. It has been known that the optimum pH for the activity of most bacterial β-1,4-mannanases is in the neutral pH range [30], while some *Bacillus* sp. β-1,4-mannanases exhibit an alkalic pH for the most activity [31,32,33]. However, fungal endo-1,4-β-mannanases often have an optimal pH for activity in the acidic range [34,35]. Although some fungal endo-1,4-β-mannanases have been reported to tolerate alkalinity up to pH 8, no alkalic fungal endo-1,4-β-mannanase has been identified [22]. On the other hand, it has been reported that the litter-decomposing basidiomycete *Coprinopsis cinerea* cultivated on barley straw solid-state medium preferred the degradation of hemicellulose rather than cellulose, and the pH in barley straw medium was over 8.5 during 6 weeks of cultivation [36]. This study aims to find a new alkalic thermophilic endo-1,4-β-mannanase from *C. cinerea* for application in some industry fields with conditions of high pH and high temperature.

## 2. Materials and Methods

### 2.1. Strains and Culture Conditions

The *C. cinerea* ATCC 56838 strain from the American Type Culture Collection (Manassas, VA, USA) was cultured for growth of fruiting bodies according to Zhang et al. [37].

*Pichia pastoris* GS115 from Invitrogen (San Diego, CA, USA) was cultivated for genetic manipulation according to the user manual provided by Invitrogen.

### 2.2. Chemicals

Locust bean gum (galactomannan polysaccharides) from seeds of *Ceratonia siliqua* (LBG, Edinburgh, UK), α-mannan from *Saccharomyces cerevisiae*, laminarin from *Laminaria digitata*, and xylan from oat spelts were ordered from Sigma (St. Louis, MO, USA). Avicel cellulose was ordered from TCI (Tokyo, Japan).

### 2.3. Analysis of the Sequence and Structure of CcMan5C

Protein sequences for all GH5_family β-mannanases in the CAZy database were retrieved from the National Center for Biotechnology Information (NCBI) (https://www.ncbi.nlm.nih.gov/, accessed on 30 September 2020). A phylogenetic tree was constructed with the MEGA7.0 software (Mega Limited, Auckland, New Zealand) using the neighbor-joining method. The conserved domain of CcMan5C was analyzed using the NCBI Conserved Domain Database. The three-dimensional structure of CcMan5C was predicted using the I-TASSER (Iterative Threading ASSEmbly Refinement, https://zhanglab.ccmb.med.umich.edu/I-TASSER/, accessed on 26 February 2023) [38].

### 2.4. Cloning and Expression of CcMan5C

Total RNA was extracted from fruiting bodies of *C. cinerea* ATCC 56838 with a Total RNA Extractor (TRIzol) (Sangon, Shanghai, China), and the total RNA was treated to remove genomic DNA with gDNA wiper Mix (Vazyme, Nanjing, China) and then used as a template to synthesize cDNA by qPCR with a HiScript^®^ II qRT SuperMix II. (Vazyme, Nanjing, China). The cDNA encoding the mature β-mannanase CcMan5C (*mCcman5C*) was amplified by PCR from the cDNA preparation with PrimeSTAR Mix (Takara, Kusatsu, Shiga, Japan) and a pair of primers, the forward primer with *EcoR*I cut-site (5′-AGAGAGGCTGAAGCTGAATTCGTAGGCCCTTGGGGCCAGT-3′) and the reverse primer with *Not*I cut-site (5′-TGTTCTAGAAAGCTGGCGGCCGCCCCGCGAGCCTTCATGG-3′).

According to the user manual of Invitrogen (USA), after being digested, respectively, with *EcoR*I and *Not*I, the PCR product containing *mCcman5C* was mixed with the plasmid pPICZαA (Invitrogen, San Diego, CA, USA) and ligated into the expression plasmid pPICZαAmCcMan5C with a ClonExpress^®^ Entry One Step Cloning Kit (Vazyme, Nanjing, Jiangsu, China). The plasmid pPICZαAmCcMan5C was linearized with *Pme*I and then electrophoretically transformed into *P. pastoris* spheroplasts. The positive transformants with pPICZαAmCcMan5C were selected on YPDS plates containing 100 μg mL^−1^ Zeocin (Invitrogen manual, San Diego, CA, USA) and then identified by colony PCR with 2 × Extaq Mix (Takara, Kusatsu, Shiga, Japan) and a pair of universal primers for the AOX1 insertion site (5′-GACTGGTTCCAATTGACAAGC-3′ and 5′-GCAAATGGCATTCTGACATCC-3′). The transformant cells with pPICZαAmCcMan5C were inoculated into 15 mL of BMGY medium (pH 6.0) in a 50-mL conical flask and cultivated overnight at 28 °C and 180 rpm (Invitrogen manual, San Diego, CA, USA). The cells were harvested by centrifugation and resuspended in 1 mL of autoclaved ddH_2_O, and then each 0.5 mL of the cell suspension was added to 100 mL of BMMY medium (pH 6.0) in a 500 mL conical flask for further cultivation for 5 days at 28 °C and 180 rpm (Invitrogen manual, San Diego, CA, USA). During cultivation, methanol at 0.5% of the total volume was added to the culture medium at an interval of 24 h to induce CcMan5C expression.

### 2.5. Purification and Identification of Recombinant CcMan5C

According to the user manual of Invitrogen (San Diego, CA, USA), the above culture medium was centrifuged at 4 °C, and the supernatant was collected and mixed with 2 × binding buffer and then loaded into a 10 mL ProteinIso^®^ Ni-NTA Resin column (Transgen, Beijing, China). After washing with binding buffer, the proteins were eluted from the Ni-NTA resin with the following elution gradient: 0–30 min, isocratic 95% binding buffer: 5% elution buffer; 30–60 min, isocratic 90% binding buffer: 10% elution buffer; 60–90 min, isocratic 85% binding buffer: 15% elution buffer; 90–120 min, isocratic 70% binding buffer: 30% elution buffer; 120–160 min, isocratic 0% binding buffer: 100% elution buffer.

The molecular size and purity of the recombinant CcMan5C protein eluted from the Ni-NTA resin were analyzed by sodium dodecyl sulfate-polyacrylamide gel electrophoresis (SDS-PAGE) [39]. The protein amount was detected by the Bradford method [40]. The recombinant CcMan5C protein was identified by matrix-assisted laser desorption/ionization time-of-flight mass spectrometry (MALDI-TOF/TOF-MS) at Sangon Biotech (Shanghai, China).

### 2.6. Analysis of Hydrolytic Activity

For analysis of the hydrolytic activity, 100 μL of reaction mixture containing 0.25 μg mL^−1^ CcMan5C and 2.5 mg mL^−1^ LBG in 50 mM PBS buffer (pH 8.0) was incubated at 50 °C and 800 rpm for 10 min; then, 100 μL of 3,5-Dinitrosalicylic acid (DNS) reagent [41] was added to the reaction mixture. The reaction mixture was heated at 100 °C for 10 min, cooled to room temperature and centrifuged. The supernatant was measured for the absorbance at 520 nm on a SpectraMax M2 microplate reader (Molecular Devices, San Jose, CA, USA) for the amount of reducing sugar (mannose used as a standard) released from LBG by CcMan5C. One unit of enzyme activity was defined as the amount of CcMan5C required to release 1 μmol of reducing sugar from LBG per minute.

For determination of the specificity of CcMan5C toward substrates, α-mannan, avicel, oat spelt xylan or laminarin was used, respectively, to replace LBG as a substrate to determine the hydrolytic activity as above. The analysis of the effects of pH, temperature, and LBG concentration on the hydrolytic activity of CcMan5C was performed as described above, except for the following changes. A 50 mM sodium acetate butter (pH 3.0–6.0), 50 mM potassium phosphate buffer (pH 6.0–8.0), or 50 mM Tris/HCl buffer (pH 8.0–9.0) was used instead of 50 mM potassium phosphate buffer (pH 8.0) in the reaction mixture for optimal pH for CcMan5C activity. The optimal temperature for CcMan5C activity was determined at various temperatures ranging from 20 °C to 80 °C. For analysis of the stability of CcMan5C under different pH and temperature conditions, the enzyme solution was first incubated at 20–80 °C, pH 8.0, or at 50 °C, pH 3.0–9.0 for 1 h and then mixed with LBG to react as described above. For analysis of the effect of the metal ions on the hydrolytic activity of CcMan5C, CcMan5C was first incubated with 1 mM metal ion salt or the metal ion chelator EDTA in 50 mM Tris-HCl (pH 7.0) at 50 °C for 1 h, and then mixed with 2.5 mg.mL^−1^ LBG and corresponding metal ion or EDTA in 50 mM Tris-HCl (pH 7.0) to react at 50 °C, 800 rpm for 10 min. The kinetic parameters of CcMan5C were determined using 0.25 mg.mL^−1^–5 mg.mL^−1^ LBG instead of 2.5 mg.mL^−1^ LBG. The K_m_ and V_max_ were determined by plotting the hydrolytic rate against the LBG concentration using OriginPro 8 SR0 (OriginLab Corporation, Northampton, MA, USA) [42]. For analysis of the effect of organic solvents or detergents on the hydrolytic activity of CcMan5C, 10% or 20% indicated organic solvents or 1% or 10% indicated detergents were added to the above reaction mixture.

### 2.7. Analysis of Stability of CcMan5C

CcMan5C solution or lyophilized CcMan5C was stored at 4 °C, −20 °C, or −80 °C in a refrigerator or freezer for the indicated times and then was used to determine the retained activity compared with the original enzyme sample as described as above.

## 3. Results

### 3.1. Gene Cloning, Recombinant Expression, Purification and Identification of Mannanase CcMan5C

**Figure 1:**
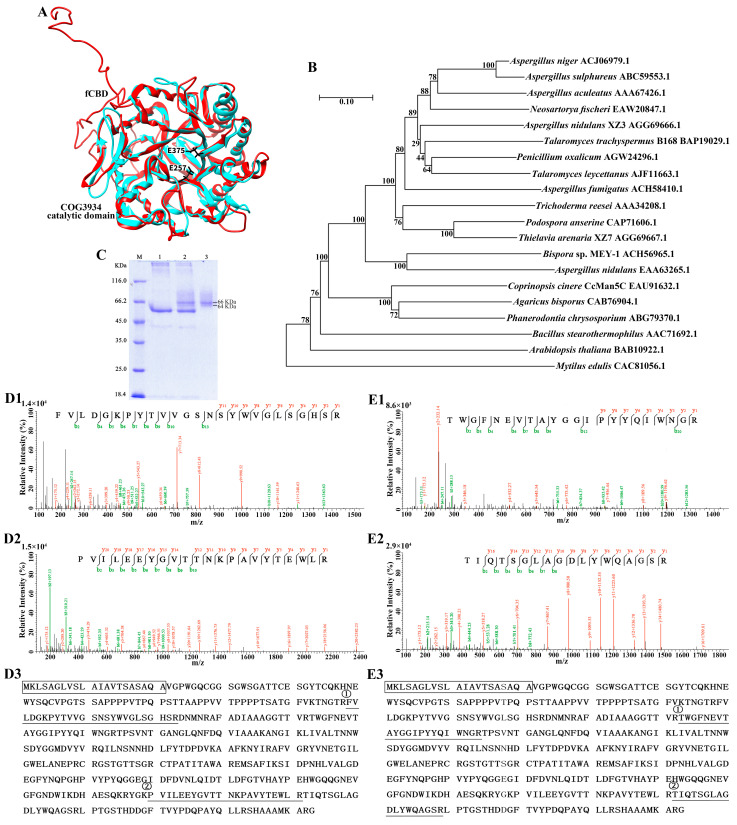
Sequence analysis, recombinant expression, and identification of endo-β-1,4-mannanase CcMan5C from *C. cinerea.* (**A**) Superimposition of the model of CcMan5C predicted by I-TASSER (red) and the closest related crystal structure of GH5 β-1,4-mannanase from *Podospora anserina* (PDB entry 3ZIZ, lake blue). The catalytic residues of CcMan5C are shown as black sticks. (**B**) Phylogenetic tree of β-mannanase homologs from different fungi, as well as the representative bacterium *Bacillus* sp., plant *Arabidopsis thaliana,* and animal *Mytilus edulis* constructed using the neighbor-joining method (1000 bootstraps) with MEGA7.0 software after ClustalW alignment. The scale bar represents 0.1 amino acid substitutions per site. Each sequence is marked with its accession No. in NCBI. (**C**) SDS-PAGE analysis. Lanes: M, standard protein markers; 1, culture medium for control stain; 2, culture medium for recombinant expression strain; 3, purified CcMan5C. (**D**) MALDI-TOF/TOF-MS spectra of the two trypsinized peptide fragments from the 66 kD band in B. Sequence 1 with a Mascot ion score of 77 and *m*/*z* 2725.35 (**D1**) and sequence 2 with a Mascot ion score of 142 and *m*/*z* 2577.3479 (**D2**) in the deduced amino acid sequence of CcMan5C (① and ②) (NCBI accession: EAU91632.1) (**D3**) are underlined, and the deduced signal peptide sequence at the N-terminus is marked by a box. (**E**) MALDI-TOF/TOF-MS spectra of the two trypsinized peptide fragments from the 64 kD band in B. Sequence 1 with a Mascot ion score of 84 and *m*/*z* 2592.2074 (**E1**) and sequence 2 with a Mascot ion score of 103 and *m*/*z* 1922.9486 (**E2**) were shown in the deduced amino acid sequence of CcMan5C (① and ②) (**E3**).

The gene sequence encoding endo-1,4-β-mannanase (Cc*man5C*) from *C. cinerea* okayama7 (#130) (GenBank accession number: XP_001830154.1) was obtained from the United States National Center for Biotechnology Information (NCBI) website (https://www.ncbi.nlm.nih.gov/, accessed on 30 September 2020). The *Ccman5C* cDNA consists of 1332 nucleic acids, encoding 443 amino acids and a stop codon (Figure 1). SignalP 5.0 server (http://www.cbs.dtu.dk/services/SignalP/, accessed on 9 October 2020) analysis predicted that the sequence of N-terminal amino acids 1–21 was a signal peptide. Conserved domain analysis at NCBI showed that CcMan5C has a COG3934 catalytic domain of endo-1,4-β-mannosidase and a fungal-type cellulose-binding domain (fCBD), belonging to GH5 family in the carbohydrate-active enzymes (CAZy) database. Amino acid sequence analysis indicated that CcMan5C showed 63.2% identity with a reported GH5 endo-1,4-β-mannanase from basidiomycete *Agaricus bisporus* (CAB76904.1) (Figure S1) [43]. The deduced amino acid sequence of CcMan5C contained two predicted conserved catalytic residues (Glu257 and Glu375). Moreover, CcMan5C had more negatively charged residues (Glu and Asp) and less polar residues (Ser and Thr) compared with the nonalkaline thermophilic endo-1,4-β-mannanases from ascomycetes (Figure S1). The model of CcMan5C was predicted by the I-TASSER server based on the closest related reported crystal structure of GH5 β-1,4-mannanase PaMan5A from *Podospora anserina* (PDB entry 3ZIZ), which has 33.4% identity with CcMan5C [44]. Although *P. anserine* PaMan5A does not contain fCBD domain, the structure of catalytic domain of CcMan5C is similar to that of PaMan5A, both of which show a (β/α)_8_-barrel fold structure belonging to COG3934 catalytic domain (Figure 1A). The two conserved catalytic glutamate residues, Glu-257 (acid-base catalytic residue) and Glu-375 (nucleophile residue), position in the active site groove of CcMan5C. A phylogenetic tree of CcMan5C and other reported GH family 5 mannanases from fungi, the bacterium *Bacillus* sp., the plant *Arabidopsis thaliana,* and the animal *Mytilus edulis* were produced with the MEGA program by the neighbor-joining method (Figure 1B), which indicated that CcMan5C from *C. cinerea* and endo-β-mannanases from the basidiomycetes *A. bisporus* and *Phanerodontia chrysosporium* form a group but are far from other fungal mannanases, especially distantly related bacterial, plant, and animal mannanases.

The 64 bp–1329 bp cDNA fragment encoding mature endo-1,4-β-mannanase (*mCcman5C*) was amplified from the mRNA extract of *C. cinerea* ATCC 56838 fruiting bodies by RT-PCR, as described in the Methods section, and inserted into the plasmid pPICZαA to generate the expression plasmid pPICZαAmCcMan5C. The plasmid pPICZαAmCcMan5C was transformed into *P. pastoris* GS115 cells to heterologously express secreted recombinant CcMan5C with a 6 × His-tag at the C-terminus.

After five days of cultivation under the introduction of methanol, the maximum endo-β-mannanase activity (40.18 U mL^−1^) against LBG in the culture medium was reached from the recombinant expression strain with pPICZαAmCcMan5C. The recombinant CcMan5C was purified from the culture medium by Ni-affinity column, the specific activity of the purified CcMan5C toward LBG was 437.2 U mg^−1^ protein, and the purification fold was 59.0, with a yield of 84.9%.

The purified recombinant CcMan5C by Ni-affinity chromatography exhibited two bands of proteins, 66 kDa and 64 kDa, on SDS-PAGE (Figure 1C). MALDI-TOF/TOF-MS analysis showed that the two partial amino acid sequences of trypsinized protein fragments in each band were consistent with CcMan5C, so both protein bands were characterized as CcMan5C (Figure 1D,E). The heterogeneity of purified CcMan5C may be due to the glycosylation of the recombinant protein in yeast *P. pastoris* [45].

### 3.2. Catalytic Features of CcMan5C

As shown in Table 1, CcMan5C hydrolyzed only LBG but not α-mannan from *S. cerevisiae* or Avicel cellulose, oat spelt xylan, or laminarin from *Laminaria digitata*.

**Figure 2:**
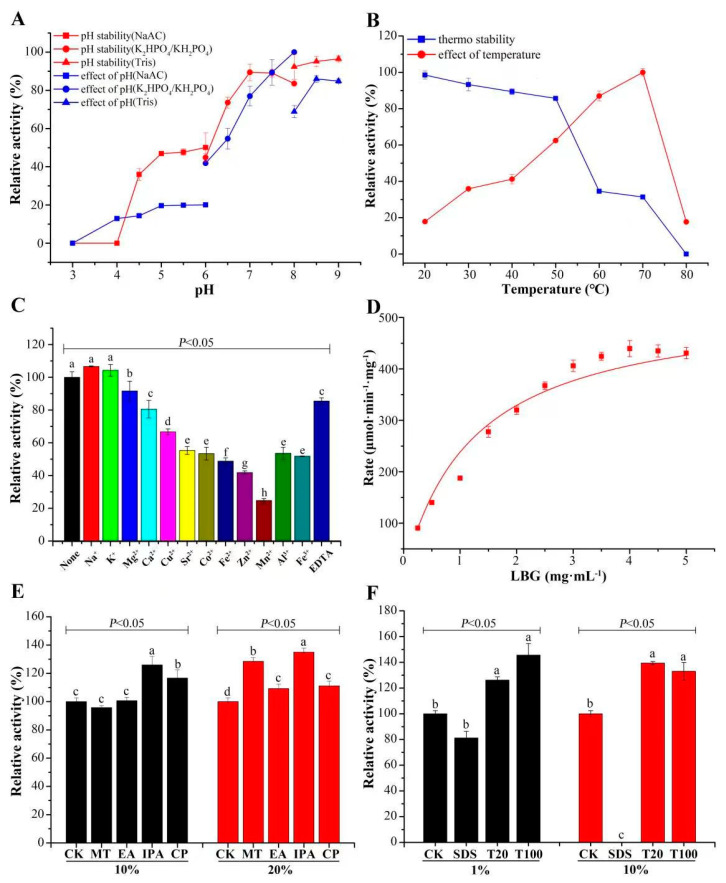
Enzyme features of CcMan5C under assay conditions. (**A**) The pH effect and pH stability of CcMan5C hydrolytic activity toward LBG at 50 °C. (**B**) The temperature effect and thermostability of CcMan5C hydrolytic activity toward LBG at pH 8.0. (**C**) Effects of metal ions and EDTA at 1 mM on CcMan5C hydrolytic activity. (**D**) The effect of LBG concentration on CcMan5C hydrolytic activity. (**E**) Effects of some organic solvents on CcMan5C hydrolytic activity. CK, control; MT, methyl alcohol; EA, ethyl alcohol; IPA, isopropyl alcohol; CP, acetone. (**F**) Effects of some detergents on CcMan5C activity toward LBG. CK, control; SDS, sodium dodecyl sulfate; T20, Tween 20; T100, Triton X-100. Data are means ± SDs (*n* = 3). The data were statistically analyzed by Duncan’s test (*p* < 0.05). The same letters indicate no significant difference (*p* > 0.05) and different letters indicate significant differences (*p* < 0.05) between hydrolytic activities of CcMan5C by Duncan’s test.

The optimal pH for CcMan5C activity toward LBG was 8.0–9.0, and CcMan5C was stable in an alkaline range of pH 8.0–9.0, retaining over 90% hydrolytic activity after 1 h of incubation in Tris-HCl buffer (pH 8.0–9.0) (Figure 2A).

The optimal temperature for CcMan5C activity toward LBG was 70 °C, and CcMan5C was stable in a range from 20 to 50 °C, retaining over 80% of its activity after 1 h of incubation at 20–50 °C (Figure 2B). Therefore, CcMan5C is an alkalic thermophilic β-1,4-mannanase.

CcMan5C activity was not significantly affected by 1 mM concentration of Na^+^ or K^+^, while the metal ion chelator EDTA at 1 mM slightly inhibited the enzyme activity (Figure 2C). This could be due to K^+^ or Na^+^ concentration in the PBS buffer because the reaction mixture sufficiently satisfied the requirement for enzyme activity and chelating monovalent metal ions by EDTA lead to decrease in enzyme activity. Furthermore, the enzyme activity was inhibited in the presence of Mg^2+^, Ca^2+^, Cu^2+^, Co^2+^, Fe^2+^, Zn^2+^, Mn^2+^, Al^3+^, and Fe^3+^ at 1 mM, which might interfere with the interaction between CcMan5C and monovalent metal ions (Figure 2C).

Different concentrations of LBG affected CcMan5C activity, and CcMan5C showed a K_m_ of 1.23 mg mL^−1^ and V_max_ of 531.77 μmol min^−1^ mg^−1^ for LBG as a substrate (Figure 2D).

The organic solvents methanol, ethanol, isopropanol, and acetone at concentrations of 10% or 20% did not inhibit CcMan5C activity toward LBG; in contrast, 10% or 20% isopropanol and acetone even enhanced CcMan5C activity by 9.20–34.98% compared with the control lacking organic solvents (Figure 2E). This indicates that CcMan5C activity tolerated the presence of organic solvents and was even stimulated by some organic solvents.

As shown in Figure 2F, 1% or 10% SDS inhibited CcMan5C activity, whereas 1% or 10% Tween-20 and Triton X-100 enhanced CcMan5C activity by 26.2%–45.6% compared with the control lacking detergents. This suggests that CcMan5C activity tolerated detergents such as Tween-20 and Triton X-100 and was even stimulated by Tween-20 and Triton X-100.

### 3.3. Storage Stability of CcMan5C

**Figure 3:**
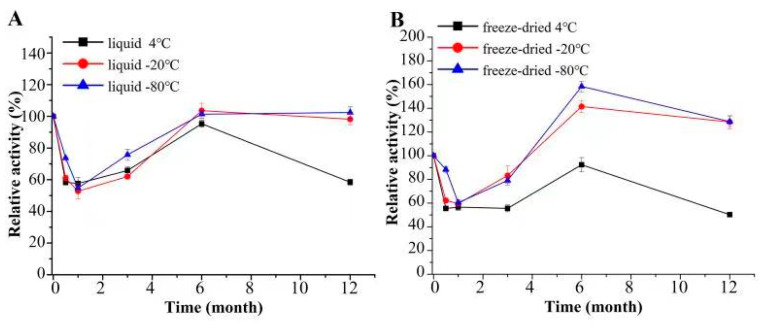
The storage stability of CcMan5C solution (**A**) or lyophilized CcMan5C (**B**).

As shown in Figure 3A,B, the CcMan5C solution stored at −20 °C or −80 °C in a freezer for the first three months showed a slight reduction in activity, while CcMan5C solution stored at −20 °C or −80 °C in a freezer for six months or even 12 months showed almost similar activity to the original enzyme solution. The lyophilized CcMan5C stored at −20 °C or −80 °C in a freezer for the first three months also showed a slight reduction in activity, whereas lyophilized CcMan5C stored at −20 °C or −80 °C in a freezer for six months showed 40% and 60% increased activity, respectively. The lyophilized CcMan5C stored at −20 °C or −80 °C in a freezer for 12 months still showed 28% increased activity compared to the original enzyme. In addition, when CcMan5C solution or lyophilized CcMan5C was stored at 4 °C in a refrigerator for 12 months, they always retained more than 50% of the activity compared with the original enzyme. These data indicated that CcMan5C possessed high storage stability.

## 4. Discussion

This study explored a novel β-mannanase, CcMan5C, from the basidiomycete *C. cinerea*, which exhibited distinctive catalytic features that were different from previously reported β-mannases in the following ways. (1) CcMan5C was the first reported fungal β-mannase with an optimal alkalic pH of 8.0–9.0 for hydrolytic activity and retained over 90% hydrolytic activity after 1 h of incubation at pH 7.0–9.0. To date, known fungal β-mannase showed an acidic optimal pH for activity [34,35], although some of the fungal endo-β-mannanases could tolerate alkalinity up to pH 8.0 [22]. For example, a β-mannanase Man5XZ7 from thermophilic fungus *Thielavia arenaria* XZ7 was optimally active at pH5.0, but it only had 25.6% activity at pH8.0–9.0 [46]. (2) CcMan5C showed an optimal temperature of 70 °C for hydrolytic activity and retained over 80% of its activity after 1 h of incubation at 50 °C. Microbial mannanases have been shown to work at different temperatures, ranging from 37 °C to 70 °C [23,24,25,31,32,47,48,49,50,51,52,53,54]. Several bacterial mannanases have displayed thermostable properties, rising up to 93 °C [23,24,25]. Some fungal acidic β-mannanases were optimally active at up to 75 °C or above [46,55,56,57,58,59]. CcMan5C is the first reported fungal alkalic β-mannanase with optimal temperature of 70 °C. (3) The organic solvents methanol, ethanol, isopropanol, and acetone at concentrations of 10% or 20% did not inhibit CcMan5C activity, while 10% or 20% isopropanol and acetone even enhanced CcMan5C activity by 9.20–34.98%. Furthermore, CcMan5C tolerated detergents such as Tween 20 and Triton X-100, and its activity was even enhanced to 26.2–45.6% by 1% or 10% Tween 20 and Triton X-100. However, most microbial β-mannanases showed a reduction to a certain extent in activity by adding organic solvents such as methanol, ethanol, and acetone [6,48,50,51], except for an endo-1,4-β-mannonase from *Bacillus pumilus* GBSW19 that showed enhancing activity by adding low concentrations of 10% isopropanol and acetone, as well as 20% ethanol [47]. Some microbial β-mannanases showed an inhibition in activity by the addition of detergents such as SDS, Tween-20 and Triton X-100 [6,50], while others showed resistance to these detergents and even an increase in activity by the addition of SDS [32,52]. (4) Furthermore, CcMan5C solution or lyophilized CcMan5C exhibited high storage stability. After being stored at −20 °C or −80 °C in a freezer for 12 months, CcMan5C solution showed almost unchanged activity, and lyophilized CcMan5C even showed 28% increased activity, whereas after being stored at 4 °C in a refrigerator for 12 months, both CcMan5C solution and lyophilized CcMan5C retained above 50% activity compared with the original enzyme. Some reported β-mannanases may have one or several of the above advantages, but CcMan5C appears in comparison to be a promising choice to investigate further, e.g., for applications in the detergent industry and paper and pulp industry with their distinct requirements [22,23,24,25].

Zhao et al. reported a structural analysis of alkalic β-mannanase from alkaliphilic *Bacillus* sp. N16-5 and found that the alkalic β-mannanase had more abundant surface-accessible negatively charged residues (Glu and Asp) when compared with the nonalkalic counterparts [60]. In contrast, the number of polar residues (Ser and Thr) on the surface in the alkalic β-mannanase was less than that in nonalkalic ones. They suggested that protein surfaces rich in acidic residues probably play an essential role in maintaining protein function under alkaline conditions, while an excess of polar residues on the protein surface might disturb the local stability at higher pH due to their unstable physical–chemical properties. In this study, sequence alignment showed that CcMan5C had more negatively charged residues (Glu and Asp) and less polar residues (Ser and Thr) compared to the nonalkaline thermophilic endo-1,4-β-mannanases from ascomycetes, and these acid amino acids were distributed on the protein surfaces by 3D structural analysis (data not shown), which may be responsible for the alkaline tolerance of CcMan5C. Of course, a convinced conclusion needs a high-resolution crystallographic analysis in the future.

It has been known that alkalic β-mannanases that show stability toward detergent components are used as stain removal boosters in certain laundry segments because β-mannanases hydrolyze different materials that contain β-mannan such as gums [22,23,24,25,32,52,59]. These β-mannan gums as thickeners or stabilizers are often used in barbecue sauce, ice cream, salad dressing, makeup, hair styling gels, shampoos, conditioners, and toothpaste. However, these gums, like a glue, stick easily soil particles and the fabric; therefore, it is difficult to remove it along with the dirt. β-mannanases can hydrolyze these gums, removing it from the fabric to prevent the dirt from sticking to the fabric. For application in the detergent industry, the enzyme should be thermostable, storable, and active in alkalic pH and detergents [22,23,24,25]. CcMan5C had an optimum temperature of 70 °C and an optimum pH of 8.0–9.0, showed an enhancing activity in the presence of Tween 20 and Triton X-100 (10%), and retained above 50% activity after one year of 4 °C storage. These features make CcMan5C a suitable candidate for the detergent industry.

Another potent application of alkalic β-mannanases is its potential use in enzymatic bleaching of softwood pulps. Pulp pretreatment under alkaline conditions hydrolyzes hemicelluloses covalently bound to lignin to facilitate subsequent removal of lignin. However, alkaline treatment of wood pulps leads to an environmental pollution problem. β-Mannanase as an alternate method equally facilitates lignin removal in pulp bleaching [6,22,23,24,25,32,43,46,52,55,56,57,58,59]. Softwoods, from which the majority of pulps are derived, contain about 15-20% galactomannan [22,23,24,25]. β-Mannanases specific for galactomannan constituents would be excellent candidates for use in enzymatic bleaching of softwood pulps. Moreover, pulping is best carried out at elevated temperatures, and alkalic thermophilic β-mannanase CcMan5C may offer significant advantages over mesophilic β-mannanases [22,23,24,25].

## 5. Conclusions

Endo-1,4-β-mannanase CcMan5C from *C. cinerea* belongs to the GH5 family in the carbohydrate-active enzymes (CAZy) database and contains a COG3934 catalytic domain and a fCBD domain. A phylogenetic analysis indicates that CcMan5C from *C. cinerea* and endo-β-mannanases from the basidiomycetes *A. bisporus* and *P. chrysosporium* form a group but are far from other fungal mannanases, especially distantly related bacterial, plant, and animal mannanases. The purified recombinant CcMan5C exhibited specific hydrolytic activity toward galactomannan (LBG) but not α-mannan or Avicel cellulose, oat spelt xylan, or laminarin. CcMan5C had an optimal alkalic pH of 8.0–9.0 and an optimal temperature of 70 °C for hydrolytic activity under assay conditions. CcMan5C tolerated some organic solvents (methanol, ethanol, isopropanol, and acetone) at concentrations of 10% or 20% and some detergents (Tween 20 and Triton X-100) at concentrations of 1% or 10%, and its activity was even enhanced by these organic solvents or detergents. CcMan5C solution or lyophilized CcMan5C also exhibited high storage stability at 4 °C, −20 °C, or −80 °C for 12 months. These distinctive features make CcMan5C a suitable candidate for application in the detergent industry and paper and pulp industry.

## Figures and Tables

**Table 1:** The ability of CcMan5C to hydrolyze different polysaccharide substrates.

Substrate	Major Linkage Type	Specific Activity (U mg^−1^) *
Galactomannan from *Ceratonia siliqua* Seeds (LBG)	β-D-Man-(1→4)-β-D-Man; α-D-Gal-(1→6)-β-D-Man	312.68 ± 1.83
α-Mannan from *Saccharomy cescerevisiae*	α-D-Man-(1→2)-α-D-Man; α-D-Man-(1→6)-α-D-Man	0
Avicel	β-D-Glu-(1→4)-β-D-Glu	0
Oatspelt xylan	β-D-xyl-(1→4)-β-D-xyl	0
Laminarin from *Laminaria digitate*	β-D-Glu-(1→3)-β-D-Glu; β-D-Glu-(1→6)-β-D-Glu	0

* Values represent the means ± SD (*n* = 3).

## Data Availability

Not applicable.

## Supplementary Materials

The following supporting information can be downloaded at: https://www.mdpi.com/article/10.3390/jof9030378/s1, Figure S1: Multiple sequence alignment of β-mannanases.
