# Supplementary material for: Heterologous Expression, Purification and Characterization of an Alkalic Thermophilic β-Mannanase CcMan5C from Coprinopsis cinerea
Source: J Fungi (Basel). 2023 Mar 20;9(3):378. doi: 10.3390/jof9030378 (PMC10056200; doi:10.3390/jof9030378)
Supplement: Supplementary file 1 [file jof-09-00378-s001.zip › jof-2214560-supplementary.pdf]

Supplementary Materials: Figure S1. Multiple sequence alignment of β-mannanases.

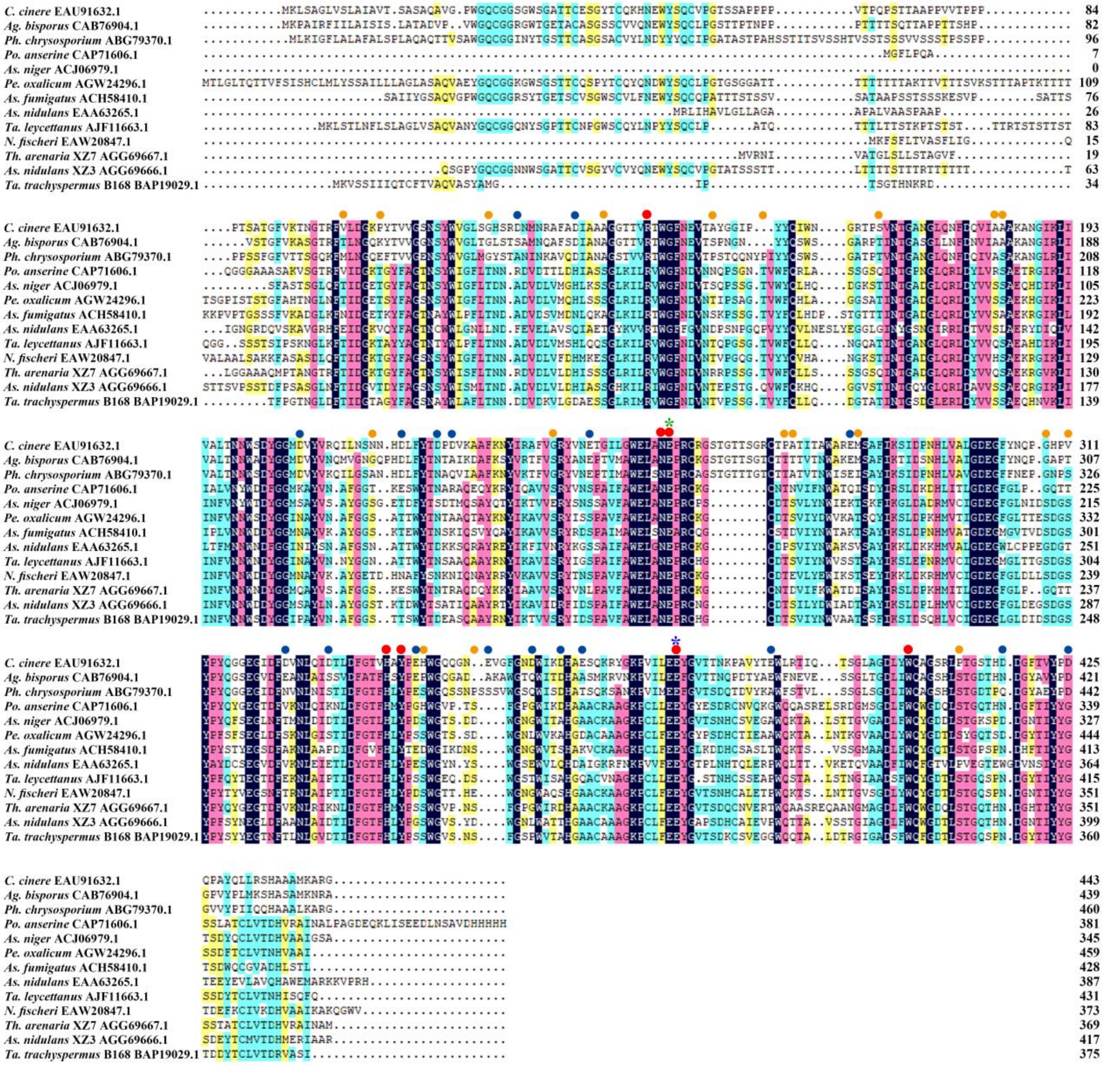

Figure S1. Multiple sequence alignment of β-mannanases from *Coprinopsis cinere*, *Agaricus bisporus* (63.2%), *Phanerodontia chrysosporium* (60.0%), *Podospora anserine* (33.4%), *Aspergillus niger* (32.1%), *Penicillium oxalicum* (39.7%), *Aspergillus fumigatus* (38.2%), *Aspergillus nidulans* (31.4%), *Talaromyces leycettanus* (39.2%), *Neosartorya fischeri* (33.3%), *Thielavia arenaria* (35.6%), *Aspergillus nidulans* XZ3 (39.5%), *Talaromyces trachyspermus* (32.3%) was done using DNAMAN software. *C. cinere* was taken as a reference. The GenBank accession numbers for amino acid sequences of fungal mannanases are presented in the figure. Identical and similar amino acids are indicated in black and red, respectively. The conserved catalytic residues are indicated by red circle dots. The negatively charged residues (Glu or Asp) in CcMan5C different from the aligning residues in β-mannanases from ascomycetes are indicated by dark blue circle dots. The non-polar residues in CcMan5C instead of polar residues (Ser or Thr) in the aligning residues in β-mannanases from ascomycetes are indicated by orange circle dots. The Glu (acid-base catalytic residue) and Glu (nucleophile residue) are indicated by green asterisk and dark blue asterisk, respectively. Percent identities of endo-1,4-β-mannanase CcMan5C from C. cinerea to other enzymes are indicated in parentheses after each species name in the legend.
